# A5 GLUTEN-SPECIFIC T CELL ACTIVATION BY MHC CLASS II EXPRESSING ORGANOID MONOLAYERS

**DOI:** 10.1093/jcag/gwab049.004

**Published:** 2022-02-21

**Authors:** S Rahmani, A Hann, H J Galipeau, F G Chirdo, T Didar, E Verdu

**Affiliations:** 1 McMaster University, Hamilton, ON, Canada; 3 Farncombe Family Digestive Health Research Institute, McMaster University, Hamilton, ON, Canada; 5 Universidad Nacional de la Plata, La Plata, Argentina

## Abstract

NOT PUBLISHED AT AUTHOR’S REQUEST

**Funding Agencies**: CIHR

